# Anti-TRAP/SSP2 monoclonal antibodies can inhibit sporozoite infection and may enhance protection of anti-CSP monoclonal antibodies

**DOI:** 10.1038/s41541-022-00480-2

**Published:** 2022-05-26

**Authors:** Brandon K. Wilder, Vladimir Vigdorovich, Sara Carbonetti, Nana Minkah, Nina Hertoghs, Andrew Raappana, Hayley Cardamone, Brian G. Oliver, Olesya Trakhimets, Sudhir Kumar, Nicholas Dambrauskas, Silvia A. Arredondo, Nelly Camargo, Annette M. Seilie, Sean C. Murphy, Stefan H. I. Kappe, D. Noah Sather

**Affiliations:** 1 Center for Global Infectious Disease Research, Seattle Children’s Research Institute, Seattle, WA USA; 2 Department of Laboratory Medicine and Pathology and Center for Emerging and Re-emerging Infectious Diseases, University of Washington, Seattle, WA USA; 3 Department of Microbiology, University of Washington, Seattle, WA USA; 4 Department of Pediatrics, University of Washington, Seattle, WA USA; 5 Department of Global Health, University of Washington, Seattle, WA USA; 6 Present Address: Vaccine and Gene Therapy Institute, Oregon Health & Science University, Beaverton, OR 97006 USA

**Keywords:** Protein vaccines, Antibodies

## Abstract

Vaccine-induced sterilizing protection from infection by *Plasmodium* parasites, the pathogens that cause malaria, will be essential in the fight against malaria as it would prevent both malaria-related disease and transmission. Stopping the relatively small number of parasites injected by the mosquito before they can migrate from the skin to the liver is an attractive means to this goal. Antibody-eliciting vaccines have been used to pursue this objective by targeting the major parasite surface protein present during this stage, the circumsporozoite protein (CSP). While CSP-based vaccines have recently had encouraging success in disease reduction, this was only achieved with extremely high antibody titers and appeared less effective for a complete block of infection (i.e., sterile protection). While such disease reduction is important, these and other results indicate that strategies focusing on CSP alone may not achieve the high levels of sterile protection needed for malaria eradication. Here, we show that monoclonal antibodies (mAbs) recognizing another sporozoite protein, TRAP/SSP2, exhibit a range of inhibitory activity and that these mAbs may augment CSP-based protection despite conferring no sterile protection on their own. Therefore, pursuing a multivalent subunit vaccine immunization is a promising strategy for improving infection-blocking malaria vaccines.

## Introduction

The last few years have marked a disheartening milestone as the first period in a generation without a reduction in the global burden of malaria^1^. The interventions that have provided much of the previous progress, such as insecticide-treated bednets and large-scale treatment programs, are highly susceptible to interruptions due to political or economic instability. This was starkly illustrated by the resurgence of malaria in Venezuela in recent years after near-elimination; and in 2020, more globally, due to interruptions in eradication efforts during the COVID-19 pandemic^1^. Therefore, it is likely that durable, infection-blocking interventions (e.g., vaccines, long-lasting mAbs, or chemoprophylactics) will be required to drive malaria to elimination.

Developing such an intervention is hampered by the complex life cycle of the *Plasmodium* parasite, which begins when an infected mosquito injects tens to hundreds of the “sporozoite” forms of the parasite into the dermis^2^. From here, sporozoites actively traverse through multiple cell types in search of an endothelial cell through which they will gain access to the blood^3^. Upon entering the bloodstream, a sporozoite is carried to the liver within minutes, where it traverses multiple cell types in the liver parenchyma and eventually establishes infection in a hepatocyte^4^. Following ~7–10 days of development and genome replication (~2 days in rodent malaria models), each successful liver-stage releases 30,000–50,000 “merozoites” that cyclically infect, replicate within, and lyse red blood cells^5,6^. It is only during this blood stage of infection when symptomatic disease occurs and is also where a subset of sexually differentiated parasite forms can be picked up by a new mosquito host to continue the transmission cycle. Each step in the infection cycle presents opportunities for intervention, although vaccines targeting the “pre-erythrocytic” stages in the skin and liver have yielded the most promising results^7^.

The most advanced pre-erythrocytic vaccine is RTS,S^8^—an antibody-eliciting subunit vaccine targeting the major sporozoite surface protein circumsporozoite protein (CSP), which has been recently recommended by the WHO^1,9^. Vaccines based on attenuated live sporozoites that arrest in the liver and function by a combination of T cells and antibodies have also demonstrated robust protection^10^. Unfortunately, despite significant efficacy from both approaches in controlled human malaria infection (CHMI) studies in malaria-naive volunteers, both vaccines have markedly reduced efficacy in field trials and have not met the goals of 75% protection against clinical disease for one year as expressed by the WHO^11^. Recent encouraging Phase II results with the R21 CSP particle in Matrix-M adjuvant do meet this goal^12^. However, protection with R21 appears dependent on high antibody titers, which would require yearly boosters that are vulnerable to interruptions, and protection is less robust against *infection*. If a vaccine or other intervention (e.g., a mAb or an injectable chemoprophylactic) is to be used as a tool to achieve malaria eradication, it will likely need at least 80% efficacy against *infection* to have a significant and sustained impact on transmission^13–15^. These realities highlight the significant room for improvement in both T cell and antibody-eliciting vaccines, with the latter more amenable to iterative improvement due to available in vitro and in vivo preclinical assays^16–19^.

Of the hundreds of proteins expressed at the sporozoite stage, at least 47 are surface-exposed^20–22^ and therefore potentially accessible to antibodies. However, few of these proteins have been rigorously investigated for their use in antibody-eliciting vaccines^23^. In addition to CSP, the thrombospondin-related anonymous protein (TRAP, also known as sporozoite surface protein 2 or SSP2) has been pursued as a vaccine candidate. Similar to CSP, TRAP is essential for sporozoite infectivity^24,25^, antibodies against it correlate with protection^26,27^ and the protein is abundant^21^ during the skin stage when parasites are particularly susceptible to antibody-mediated inhibition. The TRAP ectodomain consists of three main domains: a von Willebrand factor A-like domain (vWA), the thrombospondin repeat (TSR) domain, and a repeat region^28^. The most advanced TRAP vaccine candidate is an adenovirus/MVA-vectored vaccine that elicits strong T cell responses and has had low or mixed efficacy results in CHMI trials^29,30^ and field trials^31^, but has been improved in mice following targeting of the T cell response to the liver^32^. Antibody function in experiments involving immunization with TRAP-derived peptides have yielded mixed results ranging from significant sporozoite inhibition in vitro^33^ to no protection in vivo^34^. A combination protein TRAP/RTS,S immunization failed to show significant protection in a clinical trial^35^, while a fusion-protein approach using TRAP and CSP resulted in complete protection in a small mouse study^36^. These results using TRAP alone or in combination with CSP are difficult to interpret due to the diversity of vaccine platforms used, the possibility of immune interference in studies combining platforms, and the unclear dominance of roles for antibodies and T cells in protection^37^. Whether a more targeted TRAP antibody response could contribute to protection either alone or in combination with CSP remains poorly defined.

Here, we used both active immunization and passive transfer of mAbs raised against either *Plasmodium yoelii* (rodent malaria) or *Plasmodium falciparum* (human malaria) TRAP to more directly explore the potential efficacy of anti-TRAP antibodies. We found that anti-TRAP antibodies modestly prevent liver infection in a manner dependent on the TRAP domain recognized. Importantly, we also provide evidence that anti-TRAP antibodies with minimal protective capacity of their own may augment anti-CSP antibodies, raising their protective efficacy above 80% sterile protection. Together, these findings argue for further investigation of rationally designed multi-antigen, antibody-eliciting malaria vaccines or mAb prophylactics that target multiple antigens and might include CSP as well as non-CSP targets such as TRAP.

## Results

### PyTRAP polyclonal antibodies can prevent parasite infection of hepatocytes in vitro and in vivo

**Fig. 1:**
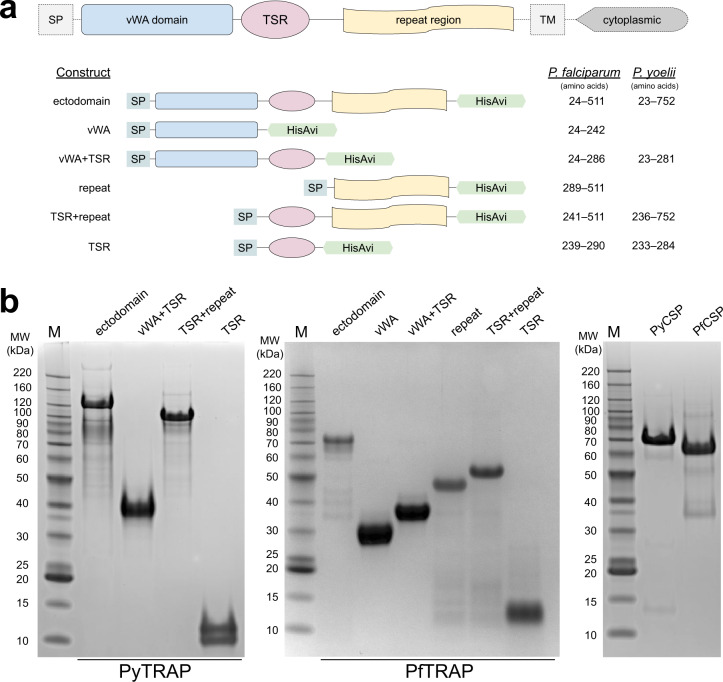
TRAP domain organization and constructs used. Ectodomain and deletion constructs for PyTRAP and PfTRAP generated using the domain boundaries (**a**) were recombinantly expressed and purified alongside the control CSP ectodomain proteins (**b**). All gels were processed in parallel. SP signal peptide, TM transmembrane region.

**Fig. 2:**
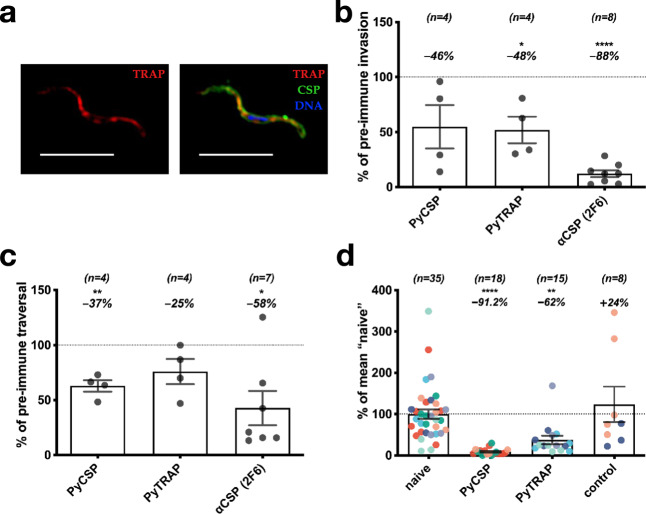
Polyclonal antibodies to PyTRAP inhibit parasite invasion, traversal, and in vivo infection. Mice were immunized three times with PyTRAP or PyCSP ectodomains. **a** Immune sera were used to verify binding to *Py* sporozoites via immunofluorescence. Shown is a representative example of fixed, permeabilized sporozoites labeled with a 1:100 dilution of polyclonal mouse serum from PyTRAP immunization. The anti-mouse IgG (anti-TRAP serum) is in the red channel shown alone on the left and in combination with anti-CSP mAb 2F6 (green channel) and a DAPI nuclear stain (blue channel); 10-µm scale bars are shown. Immune sera were then assessed for function in vitro for inhibition of invasion (**b**) and traversal (**c**). In **b** and **c**, pooled serum from cohorts of *n* = 5 mice (number of cohorts indicated above each bar) was tested in three independent assays. Each data point represents the average “% of pre-immune” invasion or traversal of these independent assays for each cohort pool. Each bar indicates the group mean, with error bars representing the standard error of the mean. Values representing percent changes from 100% (indicated by dotted lines) are shown above. Asterisks indicate a significant difference from 100% as determined by a two-tailed one-sample *t*-test. **d** Immunized mice were challenged by the bite of 15 PyGFPluc-infected mosquitoes and assessed for parasite liver burden by bioluminescent imaging. Each data point represents an individual mouse with each color corresponding to an independent immunization-challenge experiment (total number of animals shown above each bar). Each data point was normalized to the mean flux from “naive” mice within each challenge experiment, while “control” mice were an additional group immunized with HIV Env gp120 protein. Each bar indicates the group mean, with error bars representing the standard error of the mean. Values representing percent changes from 100% (indicated by a dotted line) are shown above. Asterisks indicate significance as determined by ANOVA with Kruskal–Wallis post-test. For **b**–**d**, * is *p* ≤ 0.05; ** is *p* ≤ 0.01; and **** is *p* ≤ 0.0001.

To elicit potentially functional anti-TRAP antibodies, we generated full-length ectodomains and fragments of both rodent (*P. yoelii*) and human (*P. falciparum*) malaria TRAP proteins (Fig. 1a and Suppl. Table 1) and verified their purity (Fig. 1b). Serum from mice immunized with the rodent malaria *P. yoelii* TRAP ectodomain (PyTRAP) recognized *Py* sporozoites by immunofluorescence in a pattern consistent with micronemal localization, indicating the antigenic fidelity of the recombinant protein (Fig. 2a). We further tested this serum in an inhibition of sporozoite cell traversal and invasion (ISTI) assay. Compared to control serum, anti-PyTRAP serum was able to modestly but significantly (*p* = 0.028) reduce sporozoite invasion of Hepa1-6 hepatoma cells in vitro at a level similar to serum from mice immunized with the recombinant PyCSP ectodomain, although the latter failed to reach significance (*p* = 0.106) (Fig. 2b). In contrast, sporozoite traversal of Hepa1-6 cells was not affected by anti-PyTRAP serum (*p* = 0.125), whereas anti-PyCSP serum did significantly reduce traversal (*p* = 0.0057) (Fig. 2c). The known inhibitory anti-PyCSP mAb 2F6^38,39^ reduced both inhibition and traversal in this assay, as expected (Fig. 2b, c).

PyTRAP-immunized mice were then challenged with *Py* sporozoites via mosquito bite to determine if these antibodies could function in vivo to reduce liver infection. We utilized a PyGFPluc parasite, which expresses luciferase, enabling the measurement of liver-stage parasite burden by bioluminescence imaging. Mice immunized with a nonspecific control protein (Env) showed no reduction in parasite liver-stage burden following challenge compared with naive mice. In contrast, mice immunized with the PyTRAP ectodomain showed a significant 62% reduction of parasite liver-stage burden. Mice immunized with PyCSP ectodomain showed a 91% reduction relative to naive controls (Fig. 2d). Together, these data indicate that anti-PyTRAP antibodies can function in vitro and in vivo to reduce parasite infection of hepatocytes.

### PyTRAP mAbs display a diverse array of functions in vitro and can provide additive protection to anti-CSP antibodies in vivo

**Fig. 3:**
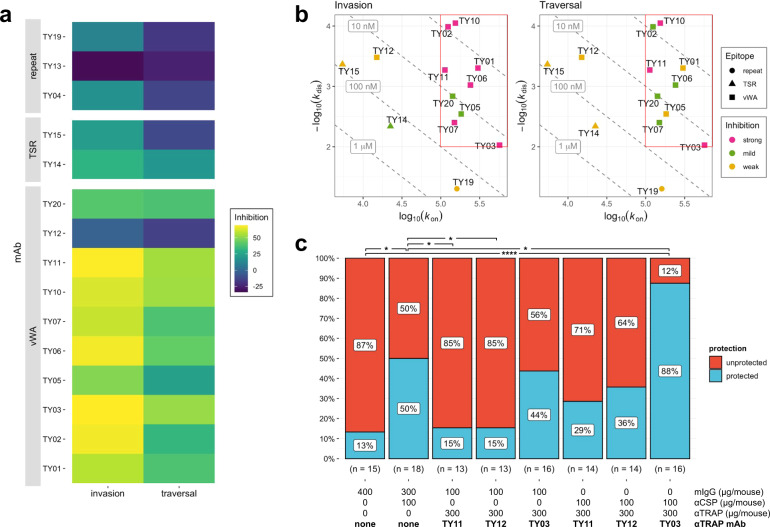
Effects of PyTRAP monoclonal antibodies on parasite activity. **a** Each mAb was assessed for in vitro function of inhibition of invasion and traversal. In each case, mean values of % inhibition (i.e., 100% – invasion or traversal value) from the 100-µg/mL mAb concentrations (bar plots with these and additional conditions shown in Suppl. Fig. 1) are represented on a color axis. **b** Binding kinetics for each mAb was measured by BLI and shown as kinetic maps with gray dashed diagonal contour lines labeled with the corresponding *K*_d_ values and symbols representing the characterized epitopes for invasion (left) and traversal (right) inhibition. Higher-affinity (i.e., those possessing lower *K*_d_ values) mAbs are closer to the upper-right corner of this plot. Symbol color coding represents “strong” inhibition for mean values ≤50%, “mild” inhibition for values ≤70%, and “weak” for mean values >70% observed at the 100-µg/mL concentration. The red box highlights the region of the kinetic plots containing the values for mAbs that showed strong inhibition in invasion and traversal assays. **c** Summarized sterile protection ratios following passive-transfer-challenge experiments (number of animals in each group is shown below the corresponding bar, individual values shown in Table 1). For **c**, * is *p* ≤ 0.05 and **** is *p* ≤ 0.0001; values reported were not adjusted for multiple comparisons due to small group sizes and limited comparisons.

Serum polyclonal antibodies, as studied above, are a mixture of many antibody specificities, making it difficult to characterize the relative contribution to the functional activity of responses directed at different domains. To enable such a characterization of the repertoire of PyTRAP-elicited antibodies, we produced a panel of 15 mAbs. When tested in ISTI at 10, 50, and 100 μg/mL, 12 of these mAbs significantly inhibited invasion or cell traversal at one or more concentrations, with mAbs TY03 and TY11 showing the most consistent and potent inhibition (Fig. 3a and Suppl. Fig. 1).

Overall, the mAbs demonstrated a wide range of binding affinities to recombinant PyTRAP (Fig. 3b and Suppl. Table 2) and recognized epitopes in the vWA, TSR, and repeat regions (Suppl. Table 3), thus covering the entire protein ectodomain. Among the 15 mAbs recovered, ten mAbs bound to the vWA domain. Six of these (TY02, TY05, TY06, TY10, TY11, and TY20) shared variable-segment assignments for both heavy and light chains, had closely related complementarity-determining-region (CDR) sequences, and had 88.4–96.7% and 93.9–96.9% sequence identity in the variable-region sequences of their heavy and light chain, respectively (Suppl. Table 4 and Suppl. Fig. 2A, B). As expected, these antibodies were functionally similar in that they bound specifically to the vWA domain (Suppl. Table 3), clustered in the same epitope bin (Suppl. Fig. 3A, B), and inhibited sporozoite infection in vitro (Fig. 3a and Suppl. Fig. 1). Two mAbs specifically recognized the TSR domain, and the remaining three mAbs bound epitopes in the repeat region (Suppl. Table 3). These non-vWA antibodies showed only modest or no sporozoite inhibition of infection in vitro (Fig. 3a). Binding interference experiments allowed for the assignment of several distinct epitope bins (Suppl. Fig. 3A, B) in addition to the one largely formed by the aforementioned group of mAbs sharing high sequence identity. This likely indicates that the mAbs in our panel bind several distant epitopes on PyTRAP. In addition, this panel of mAbs showed a wide range of binding kinetics, with all strongly inhibitory mAbs having a *k*_on_ of >10^5^ M^−1^ s^−1^ and a *k*_dis_ of <10^−2^ s^−1^ (Fig. 3b, note the red box, and Suppl. Table 4). Together, these data demonstrate that, similar to polyclonal antibodies, anti-PyTRAP mAbs can mediate anti-parasitic function in vitro, and that inhibitory function likely depends on fast and stable binding to the vWA domain. However, within the vWA domain, some epitopes show a higher correlation between binding and blocking of infection compared to others.

We next wanted to determine whether an anti-PyTRAP mAb could provide sterilizing protection in vivo on its own or in combination with an anti-CSP mAb. For this, we chose three vWA domain-binding anti-PyTRAP mAbs from distinct epitope bins: TY03 and TY11, which were the top-performing mAbs in ISTI, and TY12, which failed to demonstrate efficacy in ISTI. Neither the anti-PyTRAP mAbs nor the anti-CSP mAb showed significant binding to the mismatched Ag in vitro (Suppl. Fig. 4A, B), indicating target specificity. The anti-PyTRAP mAbs were given at 300 μg/mouse (~15 mg/kg) alone or with a partially protective dose of 100 μg/mouse (~5 mg/kg) of anti-PyCSP mAb 2F6 prior to mosquito-bite challenge^38^. As shown in Fig. 3c and Table 1, mice administered anti-PyCSP mAb 2F6 showed significant sterile protection, with 9/18 (50%) remaining blood-stage parasitemia-free, compared to 2/15 (13.3%) for mice receiving nonspecific murine IgG (*p* = 0.032; this value was not corrected for multiple comparisons due to small sample size and a small number of predefined comparisons being made). Neither TY11 nor TY12 showed any protection (2/13 or 15.4% non-infected) despite TY11 demonstrating the most robust inhibition in vitro. Administration of the mAb TY03 resulted in 7/16 mice (43.7%) remaining parasitemia-free, not reaching statistical significance. When combined with the anti-CSP mAb, only the addition of TY03 afforded significant sterile protection (87.5% or 14/16 mice) over the control group (*p* < 0.001), which, importantly, was a significant improvement over protection observed with anti-PyCSP mAb alone (*p* = 0.025; again not corrected for multiple comparisons as above). Together these data indicate that while in vitro testing of mAbs can be useful for identifying non-functional mAbs (e.g., TY12), they should be validated in vivo for function. Importantly, these data provide evidence that non-CSP antibodies may provide additive protection to anti-CSP antibodies.

**Table 1:** Combination of anti-PyCSP and anti-PyTRAP can improve sterile protection from mosquito-bite challenge.

	Sterile protection^a^ Exp 1	Sterile protection^a^ Exp 2	Sterile protection^a^ Exp 3	Sterile protection^a^ Combined	Comparison *p* value^b^ vs. 400 µg mIgG	Comparison *p* value^b^ vs. 100 µg αCSP + 300 µg mIgG
400 µg mIgG^c^	1/5 (20%)	0/5 (0%)	1/5 (20%)	2/15 (13.3%)	–	0.033
100 µg αCSP + 300 µg mIgG^c^	4/8 (50%)	3/5 (60%)	2/5 (40%)	9/18 (50%)	0.033	–
300 µg TY11 + 100 µg mIgG^c^	1/3 (33%)	0/5 (0%)	1/5 (20%)	2/13 (15.4%)	0.956	0.049
300 µg TY12 + 100 µg mIgG^c^	2/4 (50%)	0/5 (0%)	0/4 (0%)	2/13 (15.4%)	0.956	0.049
300 µg TY03 + 100 µg mIgG^c^	5/6 (83.3%)	0/5 (0%)	2/5 (40%)	7/16 (43.7%)	0.072	0.734
100 µg αCSP + 300 µg TY11	2/4 (50%)	1/5 (20%)	1/5 (20%)	4/14 (28.6%)	0.355	0.279
100 µg αCSP + 300 µg TY12	3/4 (75%)	0/5 (0%)	2/5 (40%)	5/14 (35.7%)	0.211	0.586
100 µg αCSP + 300 µg TY03	6/6 (100%)	4/5 (90%)	4/5 (80%)	14/16 (87.5%)	0.000034	0.025

^a^Sterile protection: mice that remain parasite-free (via microscopic blood-smear monitoring) throughout the experimental time course.

^b^Barnard’s exact test *p* values shown were not adjusted for multiple comparisons due to small group sizes and a small number of predefined comparisons being made.

^c^mIgG: normal mouse IgG control

Mice were injected with 100 μg/mouse of anti-CSP mAb (2F6), 300 μg/mouse of an anti-PyTRAP mAb, or a combination of both 24 h prior to challenge by five *Py*-infected mosquitoes. Where only one mAb was injected, mice were also given nonspecific mIgG to a total of 400 µg/mouse. Mice were tracked for 14 days for parasitemia by thin blood smear and those remaining parasite-free at day 14 were considered sterilely protected. The number and percentages of mice protected across three independent experiments are shown.

### Antibodies targeting the human malaria parasite *P. falciparum* TRAP can function against sporozoite invasion of hepatocytes

**Fig. 4:**
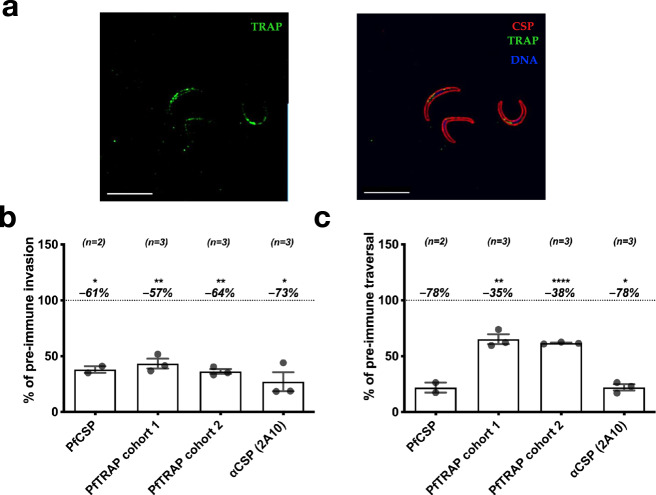
Polyclonal antibodies to PfTRAP inhibit parasite invasion and traversal in vitro. Mice were immunized three times with PfTRAP or PfCSP ectodomains. **a** Immune sera were used to verify binding to *Pf* sporozoites via immunofluorescence. Shown are fixed, permeabilized sporozoites labeled with a 1:800 dilution of polyclonal anti-PfTRAP mouse serum (followed by anti-mouse IgG secondary; green channel), fluorescently labeled anti-PfCSP monoclonal antibody 2A10 (red channel, right image) and DAPI nuclear stain (blue channel, right image); 10-µm scale bars are shown. Immune serum was then assessed for function in vitro for inhibition of invasion (**b**) and traversal (**c**). In **b** and **c**, each data point is the average “% of pre-immune” invasion or traversal from technical triplicates in independent experiments; two separate immunization experiment sets are represented as “PfTRAP cohort 1” and “PfTRAP cohort 2”. Each bar indicates the group mean, with error bars representing standard error of the mean and percent change from 100% (shown as dashed line) shown above. Asterisks indicate a significant difference from 100% as determined by a two-tailed one-sample *t*-test where * is *p* ≤ 0.05; ** is *p* ≤ 0.01; and **** is *p* ≤ 0.0001.

We next wanted to determine if antibodies directed against TRAP/SSP2 from the human malaria parasite, *P. falciparum*, could also function to prevent sporozoite infection. Serum from mice immunized with the ectodomain of *P. falciparum* TRAP (PfTRAP) was able to recognize *Pf* sporozoites in IFA (Fig. 4a) and demonstrated consistent inhibition of *Pf* sporozoite invasion in vitro at a level similar to serum from mice immunized with the ectodomain of *P. falciparum* CSP (PfCSP) (Fig. 4b). Inhibition of sporozoite traversal in vitro was more modest as compared to anti-PfCSP polyclonal serum (Fig. 4c). The known inhibitory anti-PfCSP mAb 2A10^40^ demonstrated robust inhibition of both invasion and traversal (Fig. 4b, c).

**Fig. 5:**
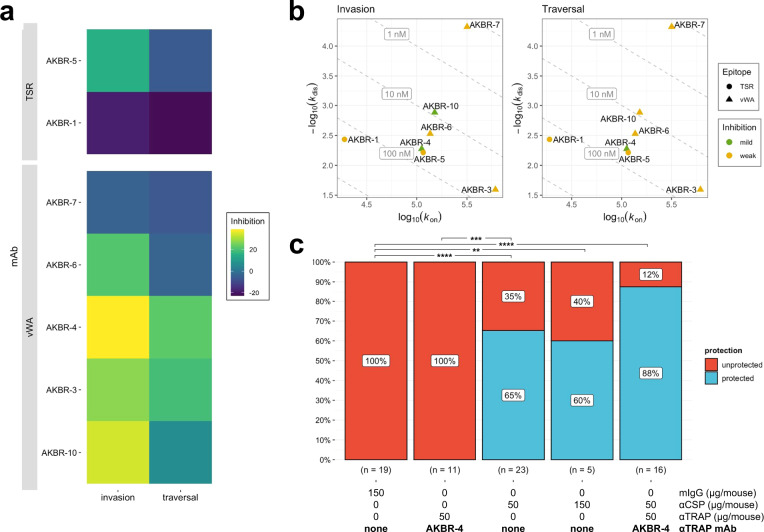
Effects of monoclonal PfTRAP antibodies on parasite activity. **a** Each mAb was assessed for in vitro function of inhibition of invasion and traversal. In each case, mean values of % inhibition (i.e., 100%—invasion or traversal value) from the 100-µg/mL mAb concentrations (bar plots with these and additional conditions shown in Suppl. Fig. 5) are represented on a color axis. **b** Binding kinetics for each mAb was measured by BLI and shown as kinetic maps with gray dashed diagonal contour lines labeled with the corresponding *K*_d_ values and symbols representing the characterized epitopes for invasion (left) and traversal (right) inhibition. Higher-affinity (i.e., those possessing lower *K*_d_ values) mAbs are closer to the upper-right corner of this plot. Symbol color coding represents “mild” inhibition for values ≤70% and “weak” for mean values >70% observed at the 100-µg/mL concentration. **c** Summarized sterile protection breakdowns following passive-transfer-challenge experiments (number of animals in each group is shown below the corresponding bar, individual values shown in Table 2). For **c**, ** is *p* ≤ 0.01, *** is *p* ≤ 0.001, and **** is *p* ≤ 0.0001; the values reported were not adjusted for multiple comparisons due to small group sizes and limited comparisons.

Using a similar approach to the anti-PyTRAP work described above, we isolated seven anti-PfTRAP mAbs from immunized mice. Of these, five mAbs recognized the vWA domain with AKBR-3, AKBR-4, and AKBR-6 likely recognizing adjacent epitopes (Suppl. Fig. 3C, D), and 2 mAbs recognized the TSR domain (Suppl. Table 3). In contrast to the high proportion of functional anti-PyTRAP mAbs (12 of 15), only two of seven anti-PfTRAP mAbs, both recognizing the vWA domain, showed any sporozoite-inhibitory function in vitro: AKBR-4 and AKBR-10. Further, only AKBR-4 demonstrated significant inhibition of both invasion and traversal (Fig. 5a and Suppl. Fig. 5), despite having unremarkable binding properties with the PfTRAP ectodomain (Fig. 5b). Surprisingly, mAb AKBR-7, which had the best binding properties of the set (*K*_d_ ~ 0.15 ± 0.04 nM, Suppl. Table 2), demonstrated the worst inhibitory properties (Fig. 5b). Similar to the case with the anti-PyTRAP mAb panel described above, our data suggest that the PfTRAP vWA domain contains epitopes exposing vulnerability to inhibition, however the lack of mAbs strongly binding to other portions of PfTRAP makes it difficult to discount the roles that these domains may play in inhibition in vivo.

### A vWA-directed anti-PfTRAP mAb increases the protection afforded by a protective CSP mAb

Because *Pf* sporozoites do not infect murine livers, the only means to test the activity of anti-*Pf* antibodies against sporozoite infection in vivo is by either challenging passively or actively immunized wild-type mice with transgenic rodent parasites expressing the *Pf* proteins of interest^41–43^ or by passive immunization of immune-deficient humanized liver mice (FRGhuHep) that can be challenged with wild-type *Pf* sporozoites^17^. We chose to utilize the latter as it is an established model of antibody-mediated protection against *Pf* infection^17,44–49^ and allows testing of any future combination of anti-*Pf* antibodies without the need for generating combinatorial transgenic parasites. In this model, humanized liver mice receive a passive transfer of antibodies and are then infected with *Pf* sporozoites via mosquito bite. Six days later, mice are injected with human red blood cells, which can then be infected by merozoites emerging from the liver, and blood-stage infection can be quantified by qRT-PCR on days 7 and 9. In this model, detection of parasites by qRT-PCR on either day 7 or 9 has proven to be a stringent and sensitive means of detecting the presence of blood-stage parasites^17,50,51^. Therefore, we define sterile protection in this model as the absence of parasites in the blood above the limit of detection at either day 7 or 9.

Using this method, we tested the ability of the anti-PfTRAP mAb AKBR-4 to provide sterile protection against *Pf* mosquito-bite infection alone or in combination with a partially protective anti-PfCSP mAb CIS43^45^. Neither the anti-PfTRAP mAb nor the anti-CSP mAb showed significant binding to the mismatched Ag in vitro (Suppl. Fig. 4C, D), indicating target specificity. We chose a dose of 50 μg/mouse (~2.5 mg/kg) for each mAb as this provides partial protection with an anti-PfCSP mAb^45^ and gives a serum concentration of ~10 μg/mL at the time of infection, which is achievable by both active vaccination and passive transfer of long-lasting mAbs^52,53^. We previously conducted passive administration, mosquito-bite challenge in two independent experiments^45^, which showed that a 50-µg/mouse dose of anti-PfCSP mAb CIS43 was protective (5/7 and 5/8 protected in each experiment), compared to control mice (0/7 and 0/7 protected). To avoid unnecessary repetition of FRGhuHep experiments, we included those cohorts in our overall analysis of mAbs in this study and conducted a third independent experiment with the control and the 50-µg/mouse dose of anti-PfCSP mAb CIS43 groups in each. In these experiments, 50 µg/mouse mAb CIS43 yielded a total of 15/23 protected (65%), which was significant compared to 0/19 of control mice protected (0%, *p* < 0.0001; Table 2 and Fig. 5c). To determine if the protection afforded by CIS43 would scale linearly with dose and possibly reach 100%, we included a group of five FRGhuHep mice in a single experiment, in which the dose was increased threefold to 150 μg/mouse. This resulted in three of five mice being protected (60%; *p* = 0.002 over control) but was not significantly different from the groups that received 50 μg/mouse.

**Table 2:** Combination of anti-PfCSP and anti-PfTRAP can improve sterile protection from mosquito-bite challenge.

	Sterile protection^a^ Exp 1	Sterile protection^a^ Exp 2	Sterile protection^a^ Exp 3	Sterile protection^a^ Combined	Comparison *p* value^b^ vs. 150 µg mIgG	Comparison *p* value^b^ vs. 50 µg αCSP
150 μg mIgG^c^	0/7 (0%)^d^	0/7 (0%)^d^	0/5 (0%)	0/19 (0%)	–	<0.0001
50 μg AKBR-4	0/6 (0%)	–	0/5 (0%)	0/11 (0%)	1	0.0002
50 μg αCSP	5/7 (71%)^d^	5/8 (63%)^d^	5/8 (63%)	15/23 (65%)	<0.0001	–
150 μg αCSP	–	3/5 (60%)	–	3/5 (60%)	0.002	0.88
50 μg AKBR-4 + 50 μg αCSP	–	6/7 (86%)	8/9 (89%)	14/16 (88%)	<0.0001	0.131

^a^Sterile protection: mice that remain parasite-free (via microscopic blood-smear monitoring) throughout the experimental time course.

^b^Barnard’s exact test *p* values shown were not adjusted for multiple comparisons due to small group sizes and a small number of predefined comparisons being made.

^c^mIgG: normal mouse IgG control

^d^indicates results previously reported in Kisalu et al.^55^.

Mice were injected with indicated doses of either nonspecific mIgG, anti-CSP mAb CIS43, anti-PfTRAP mAb AKBR-4, or a combination of anti-CSP and AKBR-4 24 h prior to challenge with five *Pf*-infected mosquitoes. Mice were injected with human red blood cells on days 5 and 6, and then blood was sampled on days 7 and 9 to detect blood-stage parasitemia by qRT-PCR. The number and percentages of mice protected across three independent experiments are shown.

On its own, passive administration of 50 μg/mouse of AKBR-4 failed to provide any sterile protection over two of these experiments (0/11, 0%). Yet, when 50 μg/mouse of AKBR-4 was combined with 50 μg/mouse of the anti-PfCSP mAb (100 μg mAb/mouse total), 14/16 (88%; *p* < 0.0001 over control) mice were sterilely protected over two independent experiments. The improvement afforded by the AKBR-4/anti-PfCSP mAb combination over the efficacy of the anti-PfCSP mAb alone trended toward but did not reach statistical significance at this group size (*p* = 0.131). Together, these results provide evidence that antibodies directed against PfTRAP may reduce *Pf* sporozoite cell traversal and invasion of hepatocytes in vitro, and potentially enhance the protection of anti-CSP mAbs when used in combination with the latter.

## Discussion

Studies examining CSP-elicited antibody responses have shown that within a polyclonal antibody population only a subset are highly potent antibody clones, and their distinguishing binding properties can be quite nuanced^39,44,45,54–58^. Understanding the characteristics associated with protection is crucial for the development of superior mAb products and vaccine immunogens, yet such studies have not been previously performed for TRAP or other non-CSP pre-erythrocytic antibody targets. Here, we show that the polyclonal antibody response to PyTRAP ectodomain can substantially reduce parasite infection of hepatocytes in vitro. We further use mAbs to conclude that this effect is likely driven by vWA and TSR-specific antibodies, although we cannot exclude the possibility of cross-reactivity with other sporozoite-expressed molecules. These findings are in line with some previous work using antibodies against TRAP protein fragments^33^, yet they contrast other observations that failed to see significant inhibition^34^. Our data with PfTRAP were more limited but the only mAb that was functional in vitro also recognized the vWA domain. Taken together, our data with polyclonal and monoclonal antibodies clearly demonstrate that TRAP is a viable antibody target and that its vWA domain contains sites of vulnerability.

Critical for any vaccine or mAb product that can be used for malaria eradication will be achieving high levels of sterile protection at sustainable antibody levels. Experience with RTS,S—which elicits extremely high peak levels of anti-CSP antibodies—as well as published data describing the activity of potent anti-CSP mAbs in animal models, suggest that increasing anti-CSP antibody titers can increase protection^45,55,59^. The first CHMI trial using passive transfer of the anti-PfCSP mAb CIS43 (also used in this study) showed that mAbs can provide sterilizing protection against *P. falciparum* mosquito-bite infection at serum concentrations between ~50–500 µg/mL^60^. However, maintenance of such high antibody titers for over a year may not be sustainable for active or passive immunization strategies. As an alternative to frequent vaccine boosting or mAb injections to sustain high titers, it may be possible to achieve high levels of protection at lower antibody titers using multivalent vaccination or multiple mAbs recognizing distinct protein targets. However, there have been no studies to date directly addressing this question, which is best examined using passive transfer of antibodies followed by mosquito-bite challenge, as done here.

Our data in the *Py* model show that an anti-PyTRAP mAb, offering no significant protection by itself, can improve protection against mosquito-bite infection of a partially protective anti-CSP mAb regimen. Our experiments using *Pf* mosquito-bite challenge in FRGhuHep mice, which received a combination of anti-PfCSP and anti-PfTRAP mAbs, did not show a statistically significant improvement over anti-PfCSP mAb treatment alone. However, the fact that this combination was the only regimen to deliver strong protection in repeated experiments, as well as the strong statistical trend toward improvement that we observed, offer support for such an approach against *P. falciparum*. Importantly, the 88% sterile protection was achieved using a low total dose of mAb (100 µg/mouse or ~5 mg/kg). This total dose of 100 µg/mouse (50 µg/mouse each of anti-PfCSP and anti-PfTRAP mAb) is expected to give a total circulating mAb concentration of ~20 µg/mL^45^ —a level that can be achieved for ~36 weeks with a single 20 mg/kg injection of long-lasting mAbs^52,60,61^ or ~4 years via active vaccination^62^. Although it remains to be seen how accurately these animal models translate to the clinic, these data suggest that reaching the 80% sterile protection threshold needed for vaccines^11^ or injectable anti-malarials^15^ that can be used as eradication tools may be achieved by targeting multiple proteins rather than by increasing the concentration of antibodies recognizing CSP alone.

Intriguingly, our data suggest that some anti-TRAP mAbs, when combined with anti-CSP mAbs, resulted in enhanced protection despite providing no statistically significant sterile protection on their own. These observations may be explained by the fact that the sterile protection readout requires the prevention of all parasites from successfully infecting the liver, effectively introducing a threshold effect. Therefore, it is possible that a weakly inhibitory mAb would have a more pronounced effect in combination with a partially protective regimen (e.g., that of a suboptimal dose of an anti-CSP mAb) than would be predicted by single-mAb experiments when using sterile protection as a readout. Additional studies clarifying the additive vs. synergistic nature of this or any multivalent approach will be needed to determine the utility of combining CSP with other immunogens but will require large group sizes and experiments designed specifically to test such hypotheses.

In summary, we present evidence that antibodies targeting TRAP may contribute to sterile protection when used in combination with anti-CSP antibodies. These findings support vaccine and mAb strategies involving multiple *Plasmodium* pre-erythrocytic-stage antigens and argue that efforts to develop a long-lasting, infection-blocking malaria intervention would greatly benefit from identifying non-CSP antibody targets that can enhance CSP-elicited protection. Although such a multivalent approach can be achieved with mAbs, it is currently limited by cost^63^. Active vaccination with multiple antigens has been hampered by challenges of generating and combining multiple protein-in-adjuvant formulations, although this may be more easily achieved by the use of mRNA-based vaccines, which have proven adept as a multi-antigen vaccine platform in preclinical studies^64,65^. Our data, which suggest that enhanced protection over CSP-only strategies is possible by way of multivalent subunit vaccination or delivery of mAbs, provide the impetus to pursue such strategies in preclinical studies that better define additive protection and identify additional targets.

## Materials and methods

### Recombinant protein production

Recombinant proteins were produced in transiently transfected suspension culture of FreeStyle 293 cells (Thermo Fisher Scientific, Waltham, MA, USA). Codon-optimized sequences encoding the ectodomains or deletion constructs of *Plasmodium falciparum* TRAP (PfTRAP), *Plasmodium yoelii* CSP (PyCSP), and *Plasmodium yoelii* TRAP (PyTRAP) were generated as fusions flanked by the tissue plasminogen activator signal sequence^66^ on the N-terminus and C-terminal 8xHis and AviTag^67^ sequences (Suppl. Table 1). Following transfection using the high-density PEI method^68^ (with 0.5 mg plasmid DNA mixed with 2 mg PEI per liter culture) and the subsequent 5-day incubation, cells were removed by centrifugation and the culture supernatants were supplemented with NaCl (+350 mM) and sodium azide (0.02%). Treated culture supernatants were passed by gravity through NiNTA agarose (Thermo), washed with Wash Buffer (10 mM Tris-HCl, pH 8, 300 mM NaCl, 10 mM imidazole), and eluted with Elution Buffer (10 mM Tris-HCl, pH 7.4, 300 mM NaCl, 200 mM imidazole). Further purification was performed by size-exclusion chromatography using a calibrated Superdex 200 (10/600) column (Cytiva, Marlborough, MA, USA). When required, site-specific biotinylation using BirA ligase (Avidity, LLC, Aurora, CO, USA), according to manufacturer’s instructions, followed by size-exclusion chromatography to remove unreacted biotin, as described above. The HIV Env gp120 control protein was produced using the FreeStyle 293 culture system described above and purified using Galanthus Nivalis Lectin agarose (Vector Laboratories, Inc, Burlingame, CA, USA), as previously described in ref. ^69^.

### Antibody cloning and production

Antibodies were cloned and produced, as previously described in ref. ^70^. Briefly, ectodomain PfTRAP and PyTRAP constructs were used as immunogens, and their biotinylated versions were used to isolate antigen-specific B cells by flow cytometry (see the sample gating strategy in Suppl. Fig. 6) using the following fluorescently labeled staining cocktail: B220-PacBlue (BioLegend cat# 103227) (BioLegend, San Diego, CA, USA), CD38-APC (BioLegend cat# 102712), IgM-FITC (BioLegend cat# 406506), and IgD-AF700 (BioLegend cat# 405730), biotinylated target complexed with streptavidin-BV785 (BioLegend cat# 405249), biotinylated decoy complexed with streptavidin-BV510 (BioLegend cat# 405233). Following co-culture with irradiated 3T3-msCD40L^71^ feeder cells in IMDM (Thermo) supplemented with 1 ng/mL IL-4 (BioLegend), 20 µg/mL LPS (Sigma-Aldrich, St. Louis, MO, USA), 50 µM β-mercaptoethanol (VWR, Radnor, PA, USA) and 1.5 µM CpG (ODN-1826) (Integrated DNA Technologies, Coralville, IA, USA), wells containing B cells producing antigen-binding IgG were identified by ELISA, immunoglobulin-encoding transcripts were amplified by RT-PCR and used for the generation of heavy- and light-chain constructs for recombinant mAb expression. The sequences were annotated using IgBLAST^72^.

To express recombinant mAbs, the heavy- and light-chain constructs were used to transfect suspension cultures of FreeStyle 293 cells (Thermo), as described above for “Recombinant protein production”. After 5 days in culture, cells were removed by centrifugation and the cultures were supplemented with NaCl (+350 mM) and sodium azide (0.02%). Treated culture supernatants were passed by gravity through Protein G resin equilibrated in Wash Buffer (10 mM HEPES, pH 7, 300 mM NaCl, 2 mM EDTA), washed with Wash Buffer, and eluted with 100 mM glycine, pH 2.7. The resulting eluates were buffer-exchanged by repeated centrifugal ultrafiltration with HBS-E (10 mM HEPES, pH 7, 150 mM NaCl, 2 mM EDTA).

### Binding properties of mAbs

Binding kinetics measurements were characterized using biolayer interferometry (BLI) measurements on an Octet QK^e^ instrument (Sartorius, Göttingen, Germany), as previously described^70^. Briefly, antibodies in culture supernatants were immobilized on anti-Mouse IgG Fc Capture biosensors and allowed to associate with antigen serially diluted (in the range of 1–1000 nM) in 10x Kinetics Buffer (10x KB: PBS + 0.1% Bovine Serum Albumin, 0.02% Tween-20, and 0.05% sodium azide) followed by dissociation in 10x KB. The resulting sensorgram data were evaluated using ForteBio Data Analysis software (version 7.0.1.5) to generate a fit to the 1:1 binding model and provide estimates for the *k*_on_ and *k*_dis_ rate constants (see sample sensorgrams and fitted curves in Suppl. Fig. 7).

The relative specificity of Ag recognition by the mAbs was assessed using biotinylated Ags (30 µg/mL, with the exception of PfCSP, which was used at 10 µg/mL) immobilized on streptavidin biosensors, and incubated with mAbs (50 µg/mL, with the exception of anti-PfCSP, which was used at 10 µg/mL) diluted in 10x KB.

Epitope bins within anti-TRAP mAb panels were assigned based on the interference patterns similar to previous work^73,74^. First, His-tagged PyTRAP or PfTRAP (30 µg/mL) was immobilized on NiNTA biosensors in HBS-NPM buffer (20 mM HEPES, pH 7, 150 mM NaCl, 1 mM MgCl_2_, 0.1 mg/mL Bovine Serum Albumin, 0.05% NaN_3_, 0.02% Tween-20). Interference for each pair of mAbs was assessed by binding the first mAb (mAb1) (50 µg/mL, except for TY14 and TY15, which were used at 100 µg/mL) to saturation before allowing the binding from the second mAb (mAb2) (50 µg/mL) to take place. The magnitude of the signal for each mAb2 binding event was corrected by subtracting the signal for the corresponding mAb1 binding step. Additionally, the signal for each mAb2 binding was collected in absence of pre-bound mAb1 (i.e., “blank” HBS-NPM buffer was used in place of mAb1 solution) and used to normalize the corrected mAb2 signal. Finally, the normalized mAb2 values were collected for each mAb1 and the resulting interference pattern sets were used to calculate the Pearson correlation coefficients using R (version 4.0.2) and plotted using the R packages pheatmap (1.0.12). Network graphs were plotted using the R package igraph (version 1.2.10) with edges connecting pairs of nodes with a Pearson correlation coefficient >0.7; and clusters of interconnected nodes are referred to as epitope bins.

### Coarse epitope mapping by ELISA

Domain specificity of the mAbs was characterized by enzyme-linked immunosorbent assay (ELISA) using TRAP ectodomain and fragments from PfTRAP and PyTRAP. Antigens were diluted in 0.1 M sodium bicarbonate (pH 9.4) and plated at 50 ng/well into Immulon 2HB plates (Thermo) followed by overnight incubation at room temperature. Plates were washed five times with wash buffer (PBS + 0.2% Tween-20) between all subsequent steps. Blocking nonspecific binding was accomplished by incubation with Block buffer (10% nonfat milk diluted in PBS + 0.3% Tween-20) for 1 h at 37 °C. Primary staining was performed using 2 ng mAb per well diluted in 0.1 mL Dilution buffer (10% nonfat milk diluted in PBS + 0.03% Tween-20), and plates were incubated for 1 h at 37 °C. Secondary staining was performed using a 1:2000 dilution of HRP Goat Anti-Mouse Ig (cat #554002) (Becton-Dickinson, Franklin Lakes, NJ, USA) prepared in the Dilution buffer, and plates were incubated for 1 h at 37 °C. Following the final wash, the plates were developed using 50 µL/well of SureBlue Reserve TMB reagent (cat #5120-0083) (SeraCare Life Sciences Inc, Milford, MA, USA) and stopped after 3 min at room temperature by the addition of 50 µL/well of 1 N sulfuric acid. Absorbance readings at 450 nm were performed using an ELx800 microplate reader (BioTek).

### Sporozoite production

For rodent parasite (*P. yoelii*), female Swiss Webster mice for parasite maintenance were purchased from Envigo (Livermore, CA, USA) and injected intraperitoneally (i.p.) with blood-stage PyGFPluc^75^. Three days later, gametocyte exflagellation was confirmed and the infected mice were used to feed female *Anopheles stephensi* mosquitoes. Fourteen to 16 days after the feed, mosquitoes were used for the mosquito-bite challenge of mice or dissected for salivary gland sporozoite isolation.

For human malaria (*P. falciparum*) experiments, *Anopheles stephensi* mosquitoes (originally from the Walter Reed Army Institute of Research) were reared following standard protocols described in the MR4 Methods in Anopheles Research manual^76^. In vitro *P. falciparum* NF54 (WT or expressing GFP and luciferase^77^) were maintained as blood-stage cultures in RPMI 1640 (25 mM HEPES, 2 mM l-glutamine) with 50 µM hypoxanthine and 10% A + human serum and O + erythrocytes in an atmosphere of 5% CO_2_, 5% O_2_, and 90% N_2_. Gametocyte cultures were initiated at 5% hematocrit with 0.8–1% mixed stage parasitemia and maintained with daily media changes for up to 17 days. To transmit parasites to mosquitoes, starved mosquitoes were allowed to feed on warm gametocyte cultures using standard membrane feeders kept at 39 °C with circulating water. Following blood-feeding, mosquitoes were maintained for up to 19 days at 27 °C, 75% humidity, and provided with 8% dextrose solution in water containing para-aminobenzoic acid (PABA). Infection prevalence and intensity were assessed by examining mosquito midguts under light microscopy on days 7–10 and mosquitoes used for either mosquito-bite infection or salivary gland sporozoite isolation 14–18 days post-feed^77^.

### Animal studies ethics statement

All procedures involving animals were performed in adherence to protocols of the Institutional Animal Care and Use Committee (IACUC) at the Seattle Children’s Research Institute.

### Mouse active immunization and challenge

To generate polyclonal serum and a source of mouse mAbs, 6- to 8-week-old BALBc/J mice were purchased from Jackson Laboratories (Bar Harbor, ME, USA) and injected intramuscularly three times at days 0, 14, and 38 using Adjuplex mixed with 20–25 µg of a target protein. Mice immunized with recombinant *Py* proteins were then challenged by the bite of 15 PyGFPluc-infected mosquitoes^38^. The proportion of mosquitoes infected with *Py* was determined by the presence of midgut oocysts on days 7–12. This proportion was used to prepare a cage with 15 infected mosquitoes per animal (i.e., if 50% of mosquitoes had oocysts and 30 mosquitoes/animals were used). These mosquitoes were then exposed to mice under ketamine/xylazine anesthesia for 10 min with the lifting of mice every minute to encourage active probing as opposed to blood-feeding. Forty-two hours later, parasite liver burden was assessed by bioluminescent imaging 42–48 h post-infection^38^. For this, mice were placed under isoflurane anesthesia and injected with 100 µL of RediJect d-luciferin (Perkin Elmer). After 5–10 min mice were transferred to the in vivo imaging system (IVIS, Caliper Life Sciences) and were imaged under isoflurane anesthesia with an exposure time of 2 min, a 10-cm diameter field of view, and a medium binning factor. Quantitation of parasite liver burden was done using Living Image 3.0 software and assessed by placing a region of interest over the mouse abdomen/liver to measure total luminescent flux in photons/second. The background signal was set to a region over the mouse pelvis and all background-subtracted data was normalized to control mice within each experiment. Mice were then immediately sacrificed and splenocytes were collected and cryopreserved for B-cell isolation and mAb production.

Mice immunized with *Pf* proteins were immunized as above with the exception that mice were additionally boosted with 20–25 µg protein (intravenous, without adjuvant) 3 days prior to sacrifice and collection and cryopreservation of splenocytes.

For both *Pf* and *Py*, serum was collected from immunized mice by collecting whole blood in BD microtainer serum tubes (Becton-Dickinson, Franklin Lakes, NJ, USA), allowing blood to clot at room temperature for at least 30 min and then centrifuged according to manufacturer’s instructions to separate serum for storage and use in in vitro assays.

### Sporozoite immunofluorescence microscopy

*Py* or *Pf* sporozoites were stained, using a “fixed-air-dried” method^78^. For this, freshly dissected *Py* or *Pf* sporozoites were fixed by resuspending in a 1.5-mL microcentrifuge tube in 4% PFA. Parasites were then pelleted at maximum speed in a microcentrifuge, resuspended in PBS to a concentration of 10^4^ sporozoites/20 µL, and air-dried by pipetting 20 µL into each well of a 12-well immunofluorescence glass slide overnight incubation. Air-dried sporozoites were then permeabilized by pipetting 0.1% Triton X-100 into each well and subsequently stained with polyclonal (serum at 1:100-1:800 dilution) or monoclonal (10 µg/mL) antibodies with three PBS washes between each step. Sporozoites were identified by co-staining with anti-CSP mAbs (at 5 µg/mL) as well as DAPI for nuclear localization. Images were acquired using an Olympus IX-70 DeltaVision deconvolution microscope at 100x magnification.

### In vitro inhibition of sporozoite traversal and invasion (ISTI)

In vitro ISTI was performed similarly for *Py* and *Pf*^79^. For these assays, freshly-isolated sporozoites were added to hepatoma cells (Hepa1-6 for *Py* and HC-04 for *Pf*) cultured in DMEM (Gibco) supplemented with 10% (v/v) FBS (Gemini Bio Products), 200 mM l-glutamine (Gibco) and 1% (v/v) Pen-strep (Gibco). These hepatoma cells were plated a day prior to infection in 96-well plates at 3 × 10^4^ cells/well. Sporozoites were added at 10^4^ spz/well in culture media in the presence of antibodies and FITC-dextran in technical duplicates or triplicates. Plates were then spun at 300×*g* for 5 min to facilitate sporozoite contact with cells and after 90 min, cells were washed with PBS, trypsinized, and transferred to a new 96-well v-bottom plate. Cells were harvested by centrifugation at 300×*g* for 5 min and were fixed and permeabilized using BD Cytofix/Cytoperm (Becton-Dickinson) according to the manufacturer’s directions. Cells were then stained with Alexa Fluor 647-labeled anti-CSP mAbs (clone 2F6 for *Py* and clone 2A10 for *Pf*), washed in PBS, and analyzed by flow cytometry. Invaded cells were identified by the presence of CSP and traversed cells by the uptake of FITC-dextran with gating set to uninfected, stained wells. Within each experimental replicate, antibody-treated wells were normalized to the invasion and traversal of wells treated with pre-immune serum or nonspecific mouse IgG, which was set to 100%.

### Anti-*Py* mAb passive transfer and challenge

Six- to eight-week-old BALBc/J mice were intravenously injected with indicated doses of mAbs 24 h prior to challenge by the bite of five PyGFPluc-infected mosquitoes following the same methods as described above for “Mouse active immunization and challenge”. Mice were followed up for infection by Giemsa-stained thin blood smear every other day from days 3–14 for identification of blood-stage parasites. Mice in which we failed to identify parasites in 40,000 red blood cells over the entire period were considered negative and sterilely protected. Control mice were administered nonspecific polyclonal mouse IgG at a dose equivalent to the highest dose in experimental groups.

### Anti-*Pf* mAb passive transfer in FRG humanized liver mice

Mice repopulated with human hepatocytes (FRGhuHep) were purchased from Yecuris, Inc. (Tualatin, OR, USA) and infected with *Pf* via mosquito bite similar to published and for *Py* experiments above^17,45^. For the challenge, indicated doses of mAb were intravenously injected into mice 24 h prior to the challenge by the bite of five *Pf*-infected mosquitoes using the same criteria and methods as above. On day 6 post-challenge, mice were intravenously injected with 400 µL of human red blood cells at 70% hematocrit. On days 7 and 9 post-infection, 100 µL of peripheral blood was collected, immediately added to 2 mL of Nuclisens lysis buffer (bioMerieux, Inc., Durham, NC, USA), incubated at room temperature for 30 min to allow for lysis, and stored at –80 °C until use for *Pf* 18S rRNA testing by quantitative reverse transcription-polymerase chain reaction (qRT-PCR). qRT-PCR was performed using previously described reagents^80^ and extraction volumes designed for the whole blood^81^. Briefly, total nucleic acids were extracted by processing 1 mL of the NucliSENS buffer-treated blood sample (containing 50 µL of mouse blood) on the EasyMag system (bioMerieux, Inc.). Extracted RNA was subsequently amplified by qRT-PCR using the AgPath-ID One-Step RT-PCR kit (Invitrogen, Waltham, MA) with a predesigned hexachlorofluorescein-labeled mouse glyceraldehyde-3-phosphate dehydrogenase (GAPDH) qRT-PCR assay (Integrated DNA Technologies) multiplexed with a pan-*Plasmodium* 18S rRNA assay. Primers/probes for the *Plasmodium* 18S rRNA assay included a pan-*Plasmodium* probe (5′[CAL Fluor Orange 560]-ACCGTCGTAATCTTAACCATAAACTA[T(BHQ1)]GCCGACTAG -[spacer C3]-3′; LCG Biosearch Technologies, Navato, CA) and flanking primers (forward, 5’-AAAGTTAAGGGAGTGAAGA-3′; reverse, 5′-AAGACTTTGATTTCTCATAAGG-3′). The following cycling conditions were used: 45 °C for 20 min, 95 °C for 15 min, and 45 cycles of 95 °C for 20 s, 50 °C for 30 s, and 60 °C for 30 s on a CFX96/1000 C real-time PCR machine (Bio-Rad, Hercules, CA). *Pf* 18S rRNA quantities were determined using a standard curve of Armored RNA calibrators. Samples were considered positive if any parasite RNA signal above the background level (i.e., a signal obtained in the reaction with no added nucleic acid) was detected in the blood.

### Statistics

Statistical analyses and plotting were carried out in Prism (version 9.2.0) (GraphPad Software, San Diego, CA, USA) or in R (version 4.0.2) using the packages Exact (version 2.1), ggpubr (version 0.4.0), and ggstatsplot (version 0.7.2). Statistical tests and outcomes are noted in the figure legend for each figure. For all tests, a *p* value of <0.05 was considered significant, and values not specifically labeled were above this threshold.

### Reporting Summary

Further information on research design is available in the Nature Research Reporting Summary linked to this article.

## Supplementary information

Supplementary Tables and Figures

REPORTING SUMMARY

## Data Availability

DNA sequences encoding the mAbs described here have been deposited in GenBank (accession numbers OK484322–OK484365).
