# Supplementary material for: Anti-TRAP/SSP2 monoclonal antibodies can inhibit sporozoite infection and may enhance protection of anti-CSP monoclonal antibodies
Source: NPJ Vaccines. 2022 May 26;7:58. doi: 10.1038/s41541-022-00480-2 (PMC9135708; doi:10.1038/s41541-022-00480-2)
Supplement: Supplementary file 2 — REPORTING SUMMARY [file 41541_2022_480_MOESM2_ESM.pdf]

nature portfolio

Corresponding author(s): D. Noah Sather, Stefan H.I. Kappe

Last updated by author(s): Oct 19, 2021

Reporting Summary

Nature Portfolio wishes to improve the reproducibility of the work that we publish. This form provides structure for consistency and transparency in reporting. For further information on Nature Portfolio policies, see our Editorial Policies and the Editorial Policy Checklist.

Statistics

For all statistical analyses, confirm that the following items are present in the figure legend, table legend, main text, or Methods section.

n/a	Confirmed	
☐	☒	The exact sample size (*n*) for each experimental group/condition, given as a discrete number and unit of measurement
☐	☒	A statement on whether measurements were taken from distinct samples or whether the same sample was measured repeatedly
☐	☒	The statistical test(s) used AND whether they are one- or two-sided<br>*Only common tests should be described solely by name; describe more complex techniques in the Methods section.*
☒	☐	A description of all covariates tested
☐	☒	A description of any assumptions or corrections, such as tests of normality and adjustment for multiple comparisons
☐	☒	A full description of the statistical parameters including central tendency (e.g. means) or other basic estimates (e.g. regression coefficient) AND variation (e.g. standard deviation) or associated estimates of uncertainty (e.g. confidence intervals)
☐	☒	For null hypothesis testing, the test statistic (e.g. *F*, *t*, *r*) with confidence intervals, effect sizes, degrees of freedom and *P* value noted<br>*Give P values as exact values whenever suitable.*
☒	☐	For Bayesian analysis, information on the choice of priors and Markov chain Monte Carlo settings
☒	☐	For hierarchical and complex designs, identification of the appropriate level for tests and full reporting of outcomes
☒	☐	Estimates of effect sizes (e.g. Cohen's *d*, Pearson's *r*), indicating how they were calculated

Our web collection on statistics for biologists contains articles on many of the points above.

Software and code

Policy information about availability of computer code

Data collection	FACSDiva flow cytometry software (version) was used in cell sorting.<br>BioTek Gen5 (version 2.06) used for ELISA data collection.<br>ForteBio Data Acquisition (version 7.0) was used for biolayer interferometry data collection.
Data analysis	Plotting and statistical analysis were performed using GraphPad Prism (version 9.2.0) and R (version 4.0.2). Flow cytometry data were analyzed using FlowJo (version 10). Biolayer interferometry data was analyzed using ForteBio Data Analysis (version 7.0).

For manuscripts utilizing custom algorithms or software that are central to the research but not yet described in published literature, software must be made available to editors and reviewers. We strongly encourage code deposition in a community repository (e.g. GitHub). See the Nature Portfolio guidelines for submitting code & software for further information.

Data

Policy information about availability of data

All manuscripts must include a data availability statement. This statement should provide the following information, where applicable:

- Accession codes, unique identifiers, or web links for publicly available datasets
- A description of any restrictions on data availability
- For clinical datasets or third party data, please ensure that the statement adheres to our policy

DNA sequences encoding the mAbs described here have been deposited in GenBank (accession numbers OK484322–OK484365).

Field-specific reporting

Please select the one below that is the best fit for your research. If you are not sure, read the appropriate sections before making your selection.

☒ Life sciences ☐ Behavioural & social sciences ☐ Ecological, evolutionary & environmental sciences

For a reference copy of the document with all sections, see nature.com/documents/nr-reporting-summary-flat.pdf

Life sciences study design

All studies must disclose on these points even when the disclosure is negative.

Sample size	Sample sizes for in vivo studies were chosen based on feasibility and based on previously published data.
Data exclusions	Data from experiments with failed controls were excluded.
Replication	In vivo experiments were carried out multiple times to ensure that different preparations of reagents or parasite stocks did not affect the statistical significance of the reported outcomes.
Randomization	Experiments were performed with cohorts of inbred lab-strain mice. No randomization was done.
Blinding	Not applicable.

Reporting for specific materials, systems and methods

We require information from authors about some types of materials, experimental systems and methods used in many studies. Here, indicate whether each material, system or method listed is relevant to your study. If you are not sure if a list item applies to your research, read the appropriate section before selecting a response.

Materials & experimental systems

n/a	Involved in the study	
☐	☒	Antibodies
☐	☒	Eukaryotic cell lines
☒	☐	Palaeontology and archaeology
☐	☒	Animals and other organisms
☒	☐	Human research participants
☒	☐	Clinical data
☒	☐	Dual use research of concern

Methods

n/a	Involved in the study	
☒	☐	ChIP-seq
☐	☒	Flow cytometry
☒	☐	MRI-based neuroimaging

Antibodies

Antibodies used	mAb 2F6 reacts with P. yoelii CSP protein, reported in public literature.<br>mAb 2A10 reacts with P. falciparum CSP protein, reported in public literature.<br>mAb CIS43 reacts with P. falciparum CSP protein, reported in public literature.<br>mAbs AKBR-1/3/4/5/6/7/10 react with P. yoelii TRAP protein, reported here and previously.<br>mAbs TY01/02/03/04/05/06/07/10/11/12/13/14/15/19/20 react with P. yoelii TRAP protein, reported here.<br>anti-B220-Pacific Blue (BioLegend, cat. # 103227)<br>CD38-APC (BioLegend, cat. # 102712)<br>IgM-FITC (BioLegend, cat. # 406506)<br>IgD-AF700 (BioLegend, cat. # 405730)
Validation	Anti-CSP and anti-TRAP antibodies were produced in-house and subjected to validation by ELISA and immunofluorescent microscopy.<br>Anti-CSP mAb CIS43 was obtained from collaborators.<br>Anti-TRAP antibodies were further validated in the course of biolayer interferometry experiments aimed at affinity measurements.<br>Commercial antibodies were validated by the manufacturer, as described in the manufacturers' websites:<br>anti-B220-Pacific Blue (https://www.biolegend.com/en-us/products/pacific-blue-anti-mouse-human-cd45r-b220-antibody-2857?GroupID=GROUP658)<br>CD38-APC (https://www.biolegend.com/en-us/products/apc-anti-mouse-cd38-antibody-180?GroupID=BLG6808)<br>IgM-FITC (https://www.biolegend.com/en-us/products/fitc-anti-mouse-igm-2334)<br>IgD-AF700 (https://www.biolegend.com/en-us/products/alexa-fluor-700-anti-mouse-igd-9571)

Eukaryotic cell lines

Policy information about cell lines

Cell line source(s)	Protein production: FreeStyle 293-F Cells (ThermoFisher)<br>ISTI: Hepa1-6 murine hepatoma and HC-04 human hepatocyte (ATCC MRA-975) line
Authentication	None of the cell lines were authenticated.
Mycoplasma contamination	The cell lines were not tested for mycoplasma contamination.
Commonly misidentified lines<br>(See ICLAC register)	None

Animals and other organisms

Policy information about studies involving animals; ARRIVE guidelines recommended for reporting animal research

Laboratory animals	Female 6–8-week-old BALB/cJ mice (Jackson Laboratories, Bar Harbor, ME, USA).
Wild animals	This study did not involve wild animals.
Field-collected samples	This study did not involve samples collected in the field.
Ethics oversight	All procedures involving animals were performed in adherence to protocols of the Institutional Animal Care and Use Committee at the Seattle Children's Research Institute.

Note that full information on the approval of the study protocol must also be provided in the manuscript.

Flow Cytometry

Plots

Confirm that:

☒ The axis labels state the marker and fluorochrome used (e.g. CD4-FITC).

☒ The axis scales are clearly visible. Include numbers along axes only for bottom left plot of group (a 'group' is an analysis of identical markers).

☒ All plots are contour plots with outliers or pseudocolor plots.

☒ A numerical value for number of cells or percentage (with statistics) is provided.

Methodology

Sample preparation	Monoclonal antibody isolation: splenocytes were prepared from immunized mice, as previously described.<br>In vitro invasion/traversal (ISTI) assays: cultured hepatocytes were used to assess invasion/traversal properties of sporozoites isolated from infected mosquito salivary glands.
Instrument	BD FACSAria II
Software	BD FACSDiva, FlowJo
Cell population abundance	Cells were single-sorted into 96-well dishes and subjected to post-sort culturing. Culture supernatants that contained antigen-specific IgG (as assessed by ELISA) were used as an indicator of clonal live B-cell cultures.
Gating strategy	Monoclonal antibody isolation: splenocytes were gated on FSC vs SSC, singlets were gated using "-H" vs "-A" plots, B220-positive populations were clearly separated, antigen-binding/decoy-free cells were identified as subpopulations emanating from the double-negative main population, CD38-positive populations were clearly separated, rare (>0.05% of total splenocytes) IgM/IgD double-negative events were harvested for subsequent culturing steps

☒ Tick this box to confirm that a figure exemplifying the gating strategy is provided in the Supplementary Information.
